# The protective effects of polysaccharide of *Atractylodes macrocephala* Koidz (PAMK) on the chicken spleen under heat stress via antagonizing apoptosis and restoring the immune function

**DOI:** 10.18632/oncotarget.19709

**Published:** 2017-07-31

**Authors:** Danning Xu, Bingxin Li, Nan Cao, Wanyan Li, Yunbo Tian, Yunmao Huang

**Affiliations:** ^1^ College of Animal Science & Technology, Zhongkai University of Agriculture and Engineering, Guangzhou 510225, China

**Keywords:** heat stress, polysaccharide of Atractylodes macrocephala koidz, apoptosis, chicken spleen, immune function

## Abstract

Heat stress can cause immune organ dysfunction and apoptosis. Polysaccharide of *Atractylodes macrocephala* Koidz may have protective effects on immune organs. In this study, we established chicken models of Polysaccharide of *Atractylodes macrocephala* Koidz-heat stress interaction and detected the oxidative index, activities of mitochondrial complexes and ATPases as well as the ultrastructure in chicken spleens. Expression levels of cytokines, mitochondrial dynamics- and apoptosis-related genes were also measured. In the result, heat stress increased the expression of interleukin 1 and tumour necrosis factor-alpha and decreased that of interleukin 2 and interferon gamma. The activities of mitochondrial complexes and ATPases were decreased and oxidative stress was induced by heat stress. Besides, expressions of the mitochondrial dynamics- and anti-apoptosis-related genes were decreased and those of pro-apoptosis-related genes were increased by heat stress. HS induced pathological changes of mitochondria and triggered apoptosis in chicken spleens. However, these adverse effects triggered by HS were remarkably alleviated in Polysaccharide of *Atractylodes macrocephala* Koidz + heat stress group. This study confirmed the protective effects of Polysaccharide of *Atractylodes macrocephala* Koidz on the chicken spleen against the heat stress and revealed its mechanism, which is that Polysaccharide of *Atractylodes macrocephala* Koidz could relieve the heat stress-induced immune dysfunction of chicken spleens via reducing oxidative stress, enhancing the mitochondria function and inhibiting apoptosis.

## INTRODUCTION

Heat stress (HS) is one of the important issues for humans and animals. It impairs the performance of farm animals, leading to economic losses globally. Previous studies have shown that poultry is very susceptible to HS [1]. HS can induce growth retardation, disrupt the intestinal barrier and damage lymphoid organs in broiler chickens [2–4]. Immune system is one of the main targets of HS-induced negative effects on the organism [5, 6]. In calf, the ability to acquire passive immunity could be negatively affected by HS [7]. In pig, HS increased the endotoxin permeability and caused immune dysfunction [8, 9]. Also, HS inhibited the production of the white blood cell and affected the immune status in laying hen [3]. Notably, the spleen, one of the immune organs, has been cited for its role in the heat stress of birds. Previous researchers observed that the weight of chicken spleen was reduced significantly under heat-stressed environment [10]. HS reduced splenic germinal centre formation in male broiler chickens [11]. Oxidative stress is often thought as one of the injurious mechanisms induced by HS. It has been indicated that HS may promote oxidative stress through blocking the antioxidant defenses. HS had been reported to increase the production of ROS and MDA in rats, and decrease the activity of superoxide dismutase (SOD) and catalase (CAT) and the total antioxidant capacity (T-AOC) in Pekin ducks [12, 13]. Notably, recent studies demonstrated that oxidative stress played a signaling role in trigging mitochondrial pathways to induce apoptotic death [14, 15]. HS had been considered to be closely related to apoptosis induced by oxidative stress in rat germ cells [16]. Also, intense HS induced apoptosis through damaging redox status, changing mitochondrial membrane potential (ΔΨm), releasing of Cyt-C, and activating caspase cascade in human umbilical vein endothelial cells (HUVEC) [17]. Since HS can induce apoptosis though mitochondrial pathway, mitochondrial functions may also have interaction with the negative effects triggered by HS. The main function of mitochondria is ATP production. This process is associated with the activities of multimeric enzyme complexes located in the inner mitochondrial membrane, such as complexes I, II and V, and ATPases [18]. Previous researchers found that ATP could significantly mediate apoptosis in human mesenchymal stem cells [19]. Additionally, evidences suggested that mitochondrial dynamics-related genes might be also associated with apoptosis. In fly, dynamin-related protein 1 (Drp1) could induce mitochondrial fragmentation and promote Drp1-dependent mitochondrial apoptosis [20]. In trophoblast BeWo cells, the increased expression of Drp1 and the decreased expressions of mitofusins (Mfn) and optic atrophy 1 (Opa1) were observed to induce the excessive of mitochondrial fragmentation and promote Drp1-dependent mitochondrial pathway apoptosis [21, 22]. In hepatocellular carcinoma, the down-regulation of Opa1 expression was also found to be involved in apoptosis [23].

*Atractylodes macrocephala* Koidz is a traditional Chinese herb medicine, and the Polysaccharide of *Atractylodes macrocephala* Koidz (PAMK) has been shown to have the ability to regulate the immune functions. In pigs, PAMK ameliorated the metabolic status and improved the immune function [24]. In mouse splenocytes, PAMK also improved the immune response though increasing the IgG and cytokines production [25]. Significantly, PAMK was also showed to have interaction with HS. In birds, PAMK significantly ameliorated oxidative injury induced by HS [26]. Besides, PAMK significantly up-regulated the expression level of Bcl-2 and down-regulated that of caspase 3 to alleviate the apoptosis induced by HS in the chicken spleen [27].

Although previous researches have studied the negative effects of HS, there are few studies in investigating the antagonistic effects of PAMK on HS in the chicken spleen discussed from the perspective of immune function and apoptosis. In the present study, we established the HS and PAMK interaction model, observed the ultrastructural changes, and examined the antioxidation, immunity and mitochondrial functions, as well as the expression levels of mitochondrial dynamics and apoptosis-related genes in chicken spleens. The aim of this study was to confirm the role of immunity dysfunction and apoptosis in HS-induced injury and investigate the mechanism of the antagonistic effect of PAMK against HS.

## RESULTS

### Ultrastructural changes

**Figure 1:**
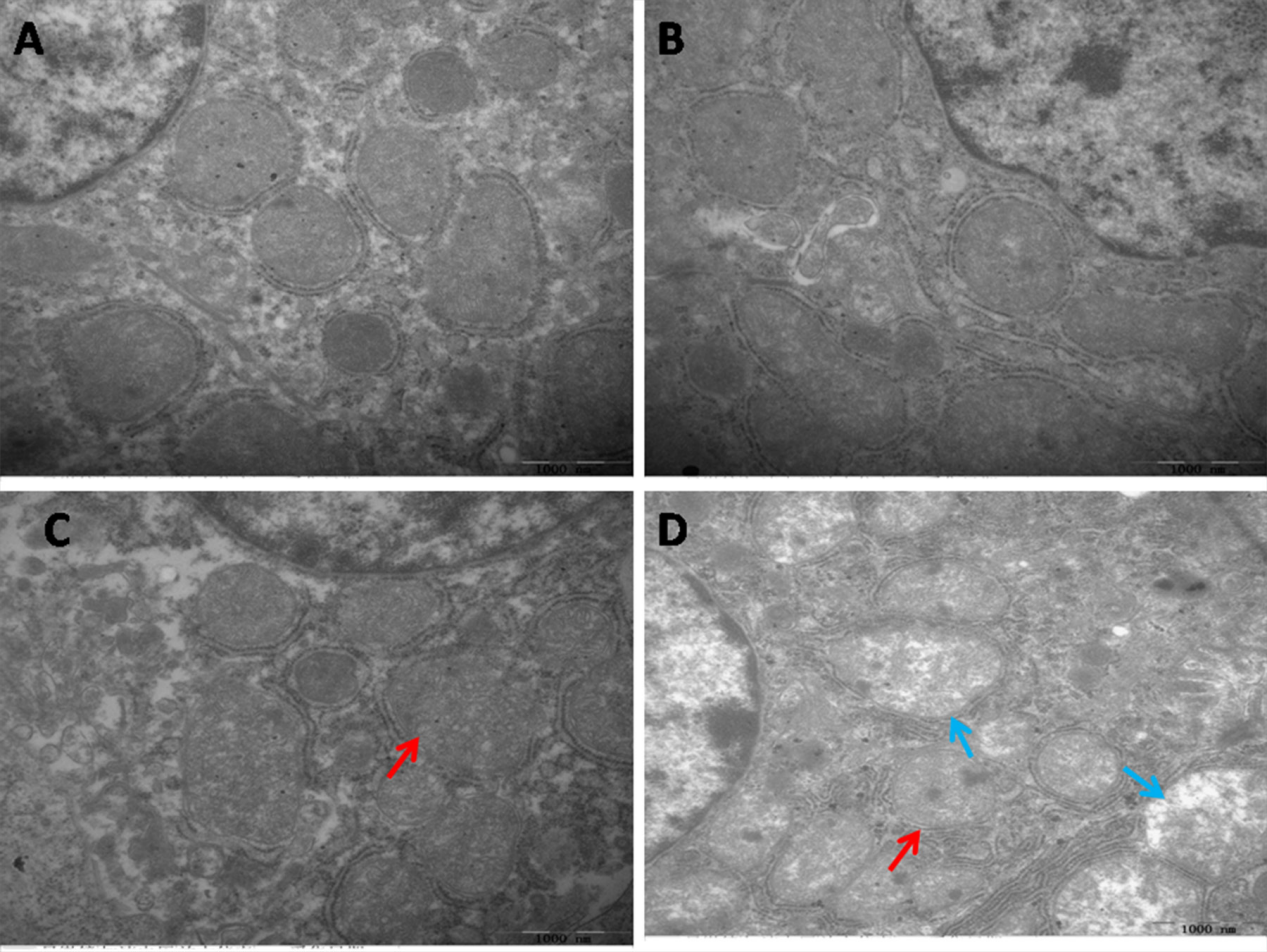
Effects of PAMK and HS on the ultrastructural changes in the chicken spleen **(A)** Spleen in the control group. **(B)** Spleen in the PAMK group. **(C)** Spleen in the PAMK+HS group. **(D)** Spleen in the HS group. The HS group showed mitochondrial vacuole (blue arrows) and mitochondrial swelling (red arrows). The pathological injuries in PAMK+HS group showed mitochondrial swelling (red arrows), but the degree of injury was less serious than that in HS group.

The results of transmission electron microscopy were showed in Figure 1. There were not any significant pathological changes appeared in the spleen of the control group and the PAMK group.

In the HS group, the cell showed severe mitochondria damage, such as mitochondria swelled, and mitochondrial cristae broken. The splenic cell of PAMK+HS group also showed mitochondria damage, but was less severe than the HS group.

### TUNEL assay

**Figure 2:**
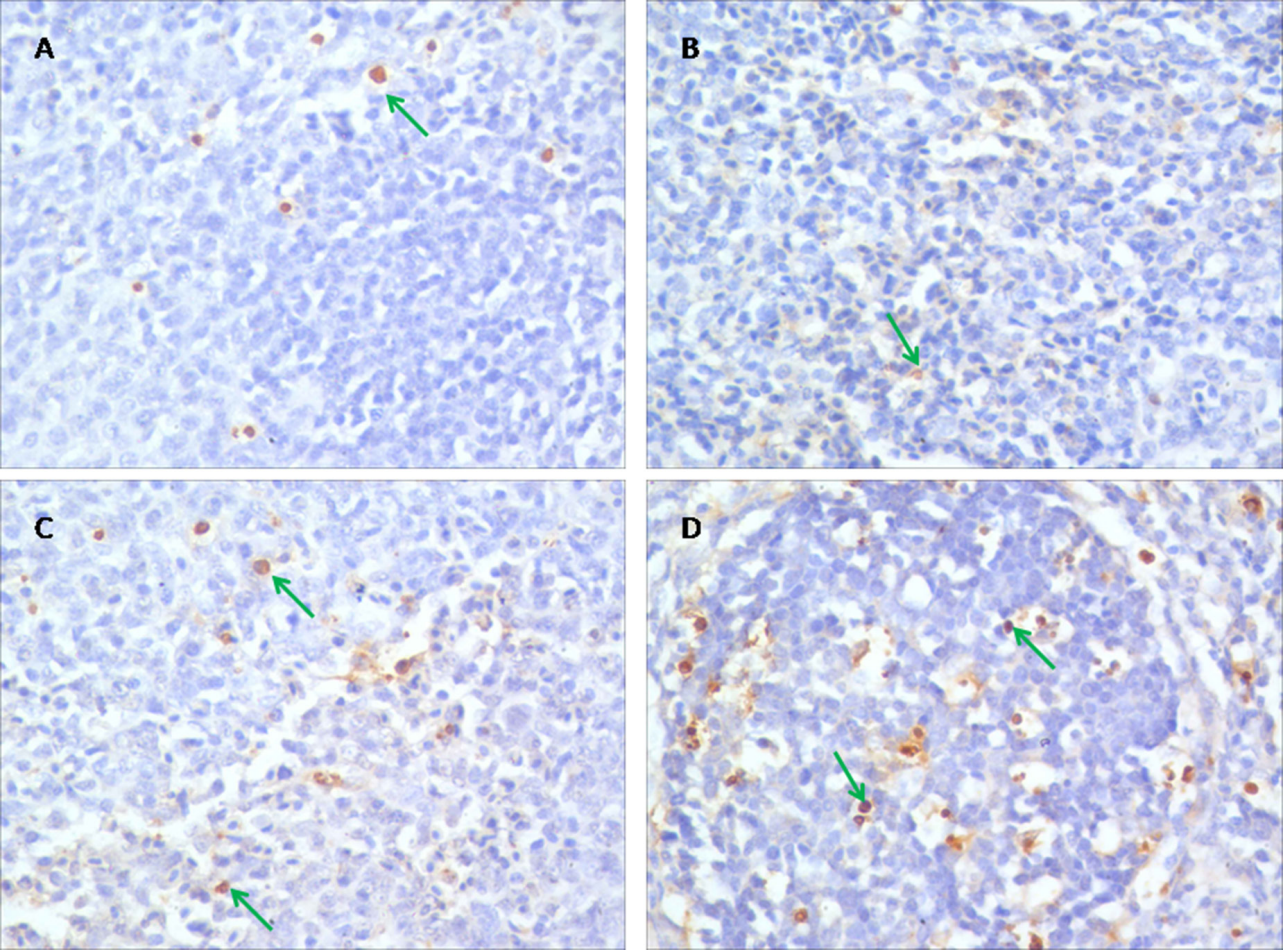
Effects of PAMK and HS on the TUNEL assay in the chicken spleen **(A)** Spleen in the control group. **(B)** Spleen in the PAMK group. **(C)** Spleen in the PAMK+HS group. **(D)** Spleen in the HS group. The magnification is × 400. Apoptotic cells had brown-stain nuclei (green arrows).

The results of TUNEL assay were showed in Figure 2. Apoptotic cells had brown-stain nuclei. There was no remarkable difference between the control group and the PAMK group (*p* > 0.05). Cells in the HS group showed a higher apoptosis rate compared with the control group, but the PAMK+HS group showed a lower apoptosis rate compared with the HS group (*p* < 0.05).

### The expressions of cytokines in the chicken spleen

**Figure 3:**
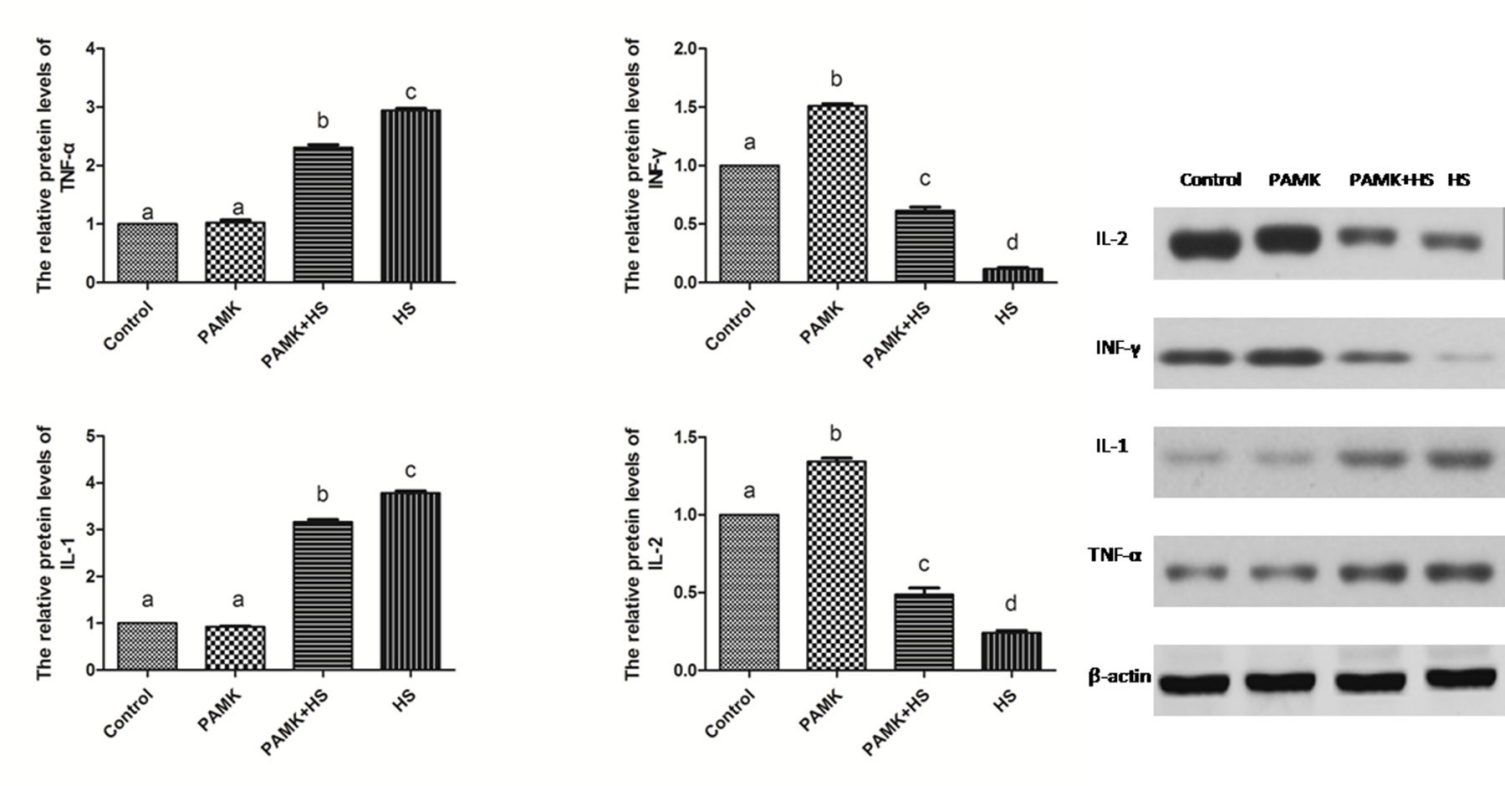
Changes of IL-1β, IL-2, INF-γ and TNF-α protein expression in the chicken spleen Each value represents the mean ± SD (*n* = 5/group). Means with the different letter represents statistically significant values (*p* < 0.05); the bars with a common letter are not significantly different (*p* > 0.05).

The protein levels of cytokines in the chicken spleen were showed in Figure 3. There were no significant changes between the control and the PAMK groups (*p* > 0.05). The relative protein levels of Interleukin 1β (IL-1β) and necrosis factor-α (TNF-α) were markedly higher and that of Interleukin 2 (IL-2) and interferon gamma (INF-γ) were lower in the HS group than those in the control group (*p* < 0.05). PAMK significantly decreased the protein levels of IL-1β and TNF-α (*p* < 0.05) and also remarkably increased that of IL-2 and IFN-γ compared with the HS group (*p* < 0.05), but did not bring them to the group levels (*p* > 0.05).

### CAT activities, T-AOC, H_2_O_2_ and ·OH inhibition contents

**Figure 4:**
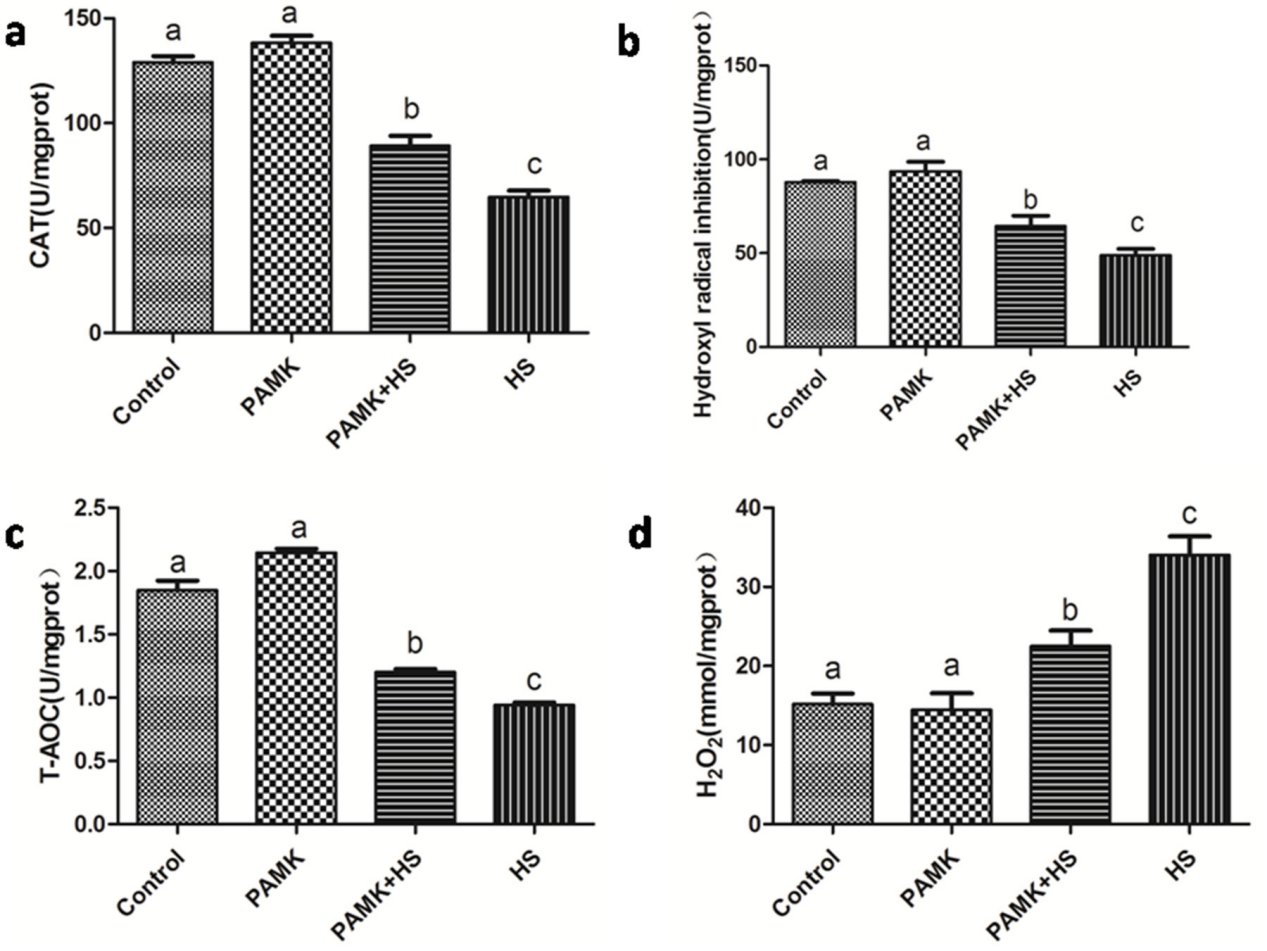
Changes of CAT activities, T-AOC, H2O2 contents and ·OH inhibition in the chicken spleen Each value represents the mean ± SD (*n* = 5/group). Means with the different letter represents statistically significant values (*p* < 0.05); the bars with a common letter are not significantly different (*p* > 0.05).

Changes of CAT activities, T-AOC, hydrogen peroxide (H_2_O_2_) content and hydroxyl radical (·OH) inhibition in the chicken spleen were showed in Figure 4. There were no significant differences between the control group and PAMK groups (*p* > 0.05). CAT activities, T-AOC and ·OH inhibition were significantly lower and the H_2_O_2_ content was higher in the HS group than that in the control group. However, the co-treatment of PAMK and HS observably alleviated those changes compared with the HS group (*p* < 0.05), but did not bring them back to the control level (*p* > 0.05).

### Activities of the mitochondrial complexes and ATPases

**Figure 5:**
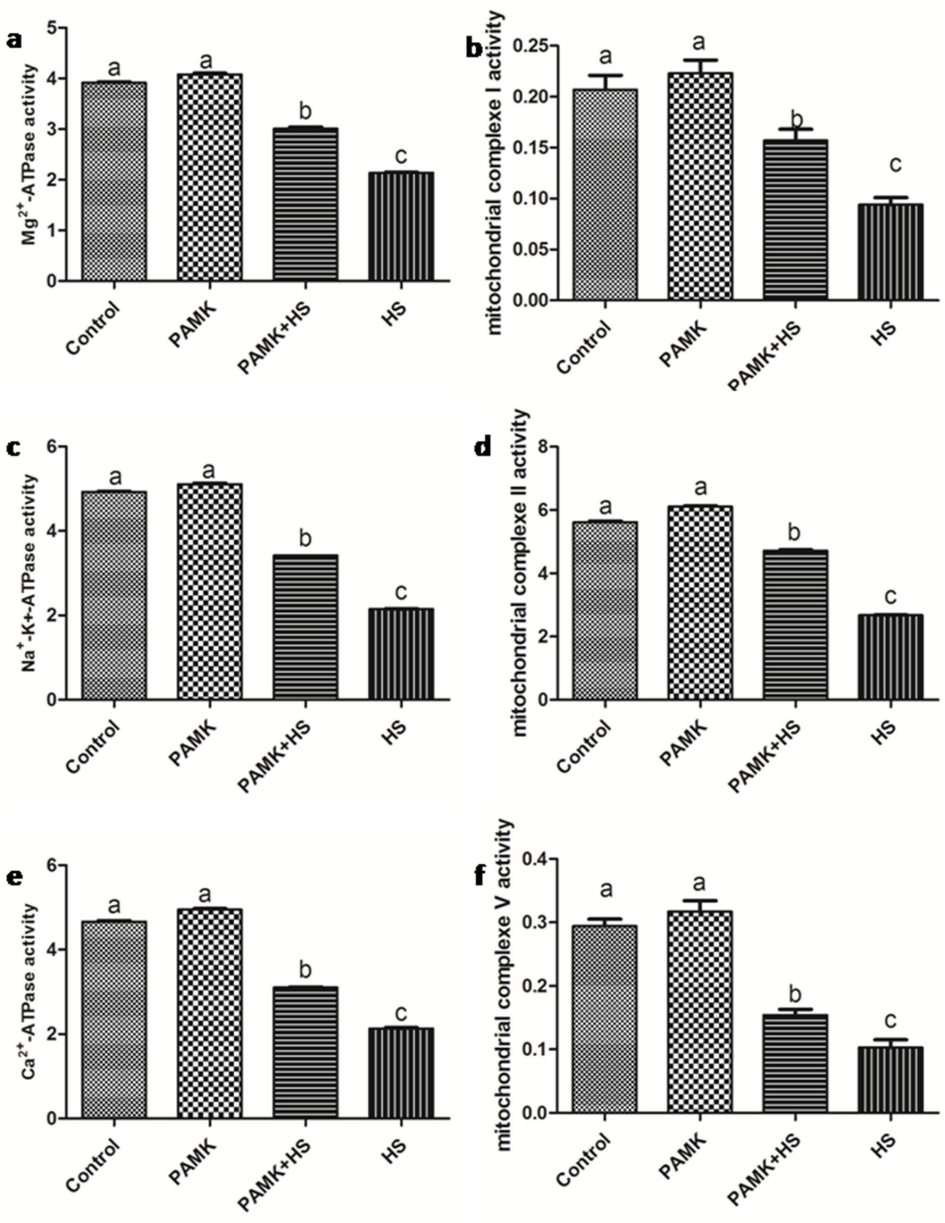
Changes of mitochondrial complexes and ATPases activities in the chicken spleen Each value represents the mean ± SD (*n* = 5/group). Means with the different letter represents statistically significant values (*p* < 0.05); the bars with a common letter are not significantly different (*p* > 0.05).

The activities of the mitochondrial complexes and ATPases in the chicken spleen were showed in Figure 5. There were no significant changes between the control group and the PAMK group (*p* > 0.05). In the HS group, the activities of mitochondrial complex I, II and V, Mg^2+^-ATPase, Na^+^-k^+^-ATPase and Ca^2+^-ATPase were significantly decreased compared with the control group (*p* < 0.05). In the PMAK+HS group, the activities of mitochondrial complexes and ATPases were observably enhanced compared with the HS group (p < 0.05), but did not reach to the control group level (p < 0.05).

### The expressions of mitochondrial dynamics- and apoptosis-related genes in the chicken spleen

**Figure 6:**
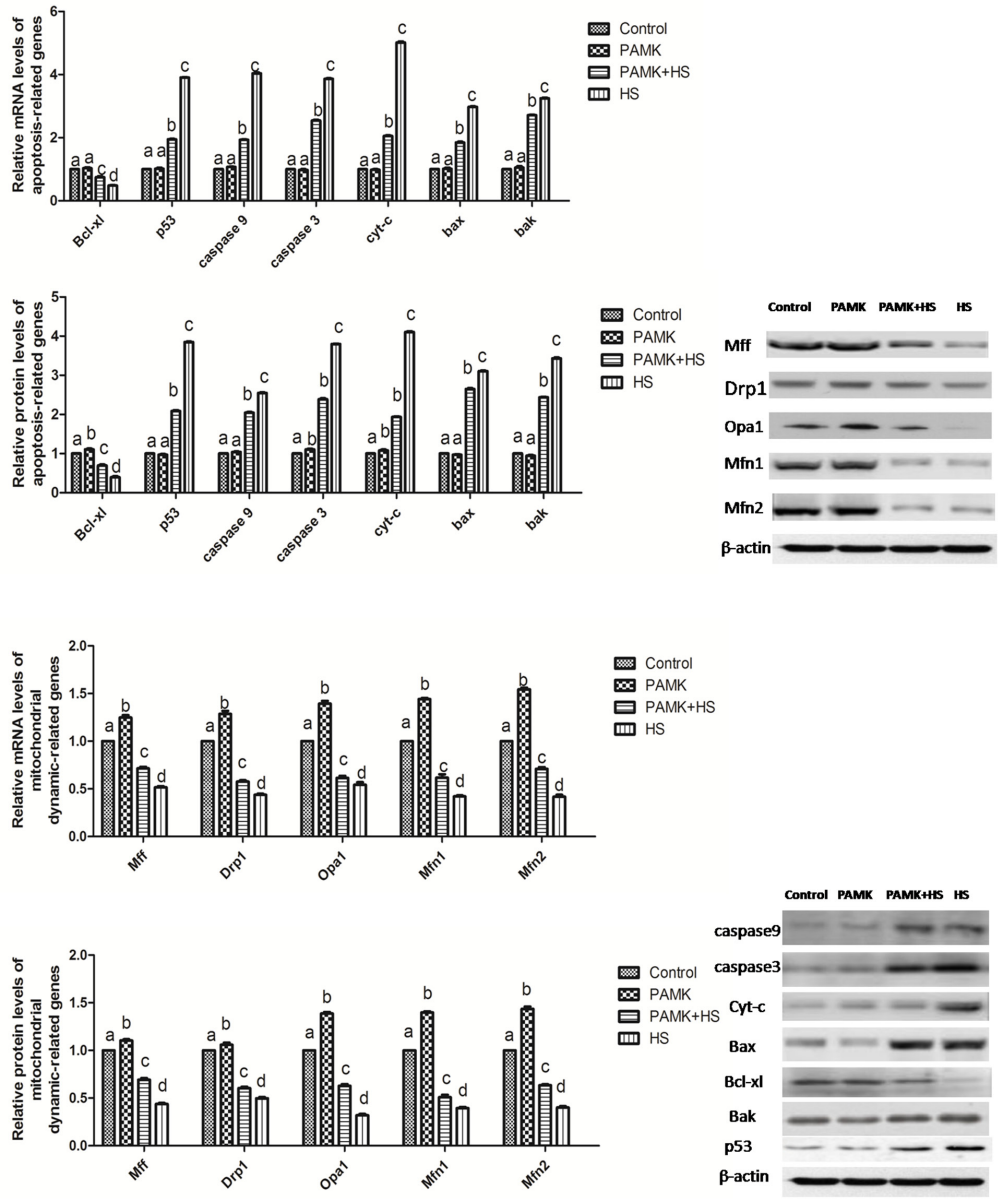
Changes of mitochondrial dynamics- and apoptosis-related genes in the chicken spleen Each value represents the mean ± SD (*n* = 5/group). Means with the different letter represents statistically significant values (*p* < 0.05); the bars with a common letter are not significantly different (*p* > 0.05).

The expressions of mitochondrial dynamics- and apoptosis-related genes in the chicken spleen were shown in Figure 6. The results showed that both mitochondrial fission- and fusion-related genes were up-regulated in the PAMK group and down-regulated in HS group compared to the control group (*p* < 0.05). Compared with the control group, the mRNA and protein levels of Cyt-c, p53, Bak and Bax were significantly increased, but that of Drp1, Mfn1, Mfn2, Mff, Opa1, and Bcl-xl were markedly decreased in the chicken spleen of the HS group (*p* < 0.05). However, in the PAMK+HS group, PAMK co-treatment significantly attenuated these changes induced by HS (*p* < 0.05).

## DISCUSSION

Studies indicated that HS has detrimental effects on humans and animals primarily though adversely influencing their body functionary, including immune function. Immune cells in various animals can be affected by heat stress. In rabbits, heat stress severely affected the immune cell function both *in vivo* and *in vitro* [28]. In chickens, lymphoid organ weights, primary and secondary antibody responses, incidences of macrophages, phagocytic ability of macrophages were all significantly reduced by HS [29]. In cows, HS increased the expressions of TNF-α and IL-1β [30, 31]. Consistent with these studies, our data showed that HS decreased the expressions of IL-2 and INF-γ, and increased that of TNF-α and IL-1 in the chicken spleen. These results suggested that the immune dysfunction was induced by HS in the chicken spleen.

In order to maintain the normal metabolism, the generation and elimination of reactive oxygen species (ROS), such as H_2_O_2_ and ·OH, usually stay in a dynamic balance. Excessive free radicals may cause damages to organisms. Evidences indicated that oxidative stress is involved in the adverse effects induced by HS. For example, liver CAT activities were decreased under HS in bream [32]. Broilers with HS treatment also showed relative higher H_2_O_2_ production compared with the regular ones [33]. In this study, we detected the activities of CAT, T-AOC, the content of H_2_O_2_ and ·OH inhibition. The results revealed that HS significantly increased the content of H_2_O_2_ and decreased the activities of CAT, T-AOC and ·OH inhibition, suggesting that the occurrence of oxidative stress was induced by HS in the chicken spleen. Additionally, previous founding has reported that oxidative stress could trigger apoptosis. Oxidative stress increased the apoptotic rate of umbilical vein endothelial cells in human [34]. Oxidative stress induced by HS also enhanced the expression of caspase 3, reduced that of bcl-2 and induced apoptosis in the chickens [26]. In agreement with these researches, our data showed that oxidative stress induced by HS significantly up-regulated the expression levels of the pro-apoptosis genes, P53, Cyt-C, Bax, Bak, Caspase 9 and Caspase 3, and down-regulated the anti-apoptosis gene, Bcl-xl. Besides, the results of transmission electron microscopy showed the typical features of pathological changes in HS group, such as mitochondrial cristae broken and chromatic agglutination. These results indicated that HS impacted the redox status, and promoted the mitochondrial apoptosis in the chicken spleen. Previous researchers found that apoptosis might have interactions with mitochondrial dynamics. Drp1 was confirmed to be required for Bax translocation to mitochondria in response to apoptosis [35]. In hepatocellular carcinoma, cell apoptosis was directly induced via targeting Opa1 to trigger mitochondrial injury [23]. In the current study, we detected the expressions of the mitochondrial dynamics-related genes, including Mff, Drp1, Opa1, Mfn1 and Mfn2. The results showed that both mitochondrial fission- and fusion-related genes were down-regulated under HS condition. It suggested that HS decreased the number of mitochondria in the chicken spleen. Besides, previous study also documented that in human mesenchymal stem cells, ATP significantly prevented serum deprivation-induced apoptosis [19], suggesting that apoptosis may be related to the generation and elimination of ATP. The processes of ATP generation and elimination are required for the activities of ATPases and mitochondrial complexes. Therefore, in the present study, we examined the activities of ATPases (Na^+^-K^+^-ATPase, Mg^2+^-ATPase and Ca^2+^ATPase) and mitochondrial complexes (mitochondrial complexes I, II and V), and found that the activities of these ATPases and mitochondrial complexes were all decreased under HS treatment. Because the main function of mitochondria is ATP production, lower ATPases activities suggested the occurrence of mitochondrial dysfunction. The decreased number and blocked function of mitochondria may induce the reduction of ATP generation and then further promote apoptosis.

Previous researchers found that PAMK can modulate the immune function. In mouse splenocytes, PAMK treatment increased the expressions of IL-2 and TNF-α [37]. In foot-and-mouth disease mice, oral administration of PAMK also promotes the immune responses [38]. Consistent with these studies, we also found that PAMK significantly enhanced the lower expressions of IL-1 and TNF-α and reduced higher levels of IL-2 and INF-γ expressions changed by HS. It indicated that PAMK can restore and improve the HS-induced immune dysfunction in the chicken spleen. Additionally, researchers also stated that PAMK can alleviate oxidative stress induced by HS. In chickens, the higher level of MDA induced by HS was remarkably reduced by PAMK [39]. In our study, the decreased activities of CAT and T-AOC induced by HS were increased under the PAMK+HS condition, as well as ·OH inhibition. The higher level of H_2_O_2_ induced by HS was also restored by the treatment of PAMK. It suggested that PAMK can relieve the HS-induced oxidative stress in the chicken spleen. Furthermore, previous studies also stated that PAMK may antagonize apoptosis in the chicken [36, 40]. In the present study, PAMK decreased the expressions of pro-apoptotic genes, and increased the expression of anti-apoptotic gene. It elucidated that PAMK has an antagonistic effect on the HS-induced apoptosis. Evidence also indicated that apoptosis pathway could be antagonized by reducing the number of mitochondria and inhibiting the function of mitochondria [41]. In our results, both mitochondrial fission- and fusion-related genes were up-regulated in the PAMK group. It might suggest that PAMK helped in a general enhancement of the mitochondrial number. Besides, we found that PAMK significantly alleviated the HS-induced changes of mitochondrial dynamics-related genes to restore the number of mitochondria and remarkably increased the activities of ATPases and mitochondrial complexes decreased by HS to recover the function of mitochondria. Moreover, We also observed that PAMK remarkably ameliorated ultrastructural damage induced by HS in chicken spleens. Therefore, we speculated that PAMK can restore the immune function, alleviate the oxidative stress, recover the number and function of mitochondria, and make the mitochondrial apoptotic pathway blocked to relieve the HS-induced apoptosis in the chicken spleen.

In conclusion, we found that HS caused the immune dysfunction and oxidative stress, and triggered apoptosis via decreasing the activities of ATPases and mitochondrial complexes and regulating the expressions of mitochondrial dynamics- and apoptosis-related genes. However, the treatment of PAMK can significantly alleviate HS-induced damage in chicken spleens.

## MATERIALS AND METHODS

### Animal care and experimental design

All procedures used in the current study were approved by the Zhongkai University of Agriculture and Engineering. Chickens (*n* = 100) were randomly divided by body weight to four groups (25 chickens per group). Each treatment was replicated five times with 5 chickens each. During the first 3 weeks, the chambers were not separated and recommended brooding temperatures were applied. The control diets consisted of a basic diet without PAMK. The PAMK supplemental diets consisted of a basic diet with the PAMK (200 mg kg^-1^). During the experimental period, consumption of feed and water were supplied ad libitum, and feed intake and body weights were recorded weekly. Clinical symptoms and mortality were also recorded. Following euthanasia with sodium pentobarbital, the spleen tissues were quickly removed for the subsequent experiments.

### Temperature and treatments

After the first 3 weeks, chickens were subjected to either a high temperature or the optimum temperature. In HS group and PAMK+HS group, the ambient temperature was set to 37±1°C. In control group and the PAMK group, the temperature was kept 23.9°C. The relative humidity was allowed to fluctuate, but not to levels below 55 %. The feed intake and body weights were recorded weekly. During the experiment, the chickens were on a lighting schedule of 24 h of light, and the average light intensity is 15 lx.

### Transmission electron microscopy

For electron microscopy, the spleen tissues (sized, 1.0 × 1.0 × 1.0 mm) were fixed immediately with 2.5 % glutaraldehyde phosphate-buffered saline (v/v, pH 7.2), post-fixed in 1 % osmium tetroxide (v/v), and stained with 4.8 % uranyl acetate. Then, the samples were dehydrated in a graded ethanol series and embedded in Epon. Ultrathin sections (≤ 90 nm) were cut, mounted on coated copper grids, washed in propyleneoxide, impregnated with epoxy resins, and post-stained with uranyl acetate, and lead citrate. Specimens were then observed under transmission electron microscopy (GEM-1200ES, Japan).

### TUNEL assay

TdT-mediated dUTP nick end labeling (TUNEL) detection of apoptotic cells was carried out with commercial cell apoptosis detection kit according to the manufacturer’s protocol (Roche Co. Ltd., China). Proteinase K was used to treat Paraffin wax-embedded tissue sections, and the endogenous peroxidase activity was inhibited with hydrogen peroxide. And the sections were incubated at 37°C for 1 h with the terminal TdT nucleotide mixture. The reaction was then stopped and the slides were rinsed with phosphate-buffered saline. Nuclear labelling was carried out with horseradish peroxidase and diaminobenzidine. Haematoxylin was used for counterstaining.

### Activity of the mitochondrial complex

The spleen tissues were homogenized in a buffer (1 mM EDTA, 0.1 M Tris–HCl,0.1 M sucrose, 5 mM KCl, pH = 7.4) by a loose-fitting Dounce (Teflon-glass) homogenizer. The homogenates were quickly transferred to Eppendorf tubes and centrifuged at 1000 × g for 10 min at 4°C. The supernatant was centrifuged at 1000 × g for 5 min and then 13,000 × g for 20 min at 4°C to precipitate the mitochondria. The mitochondria from the spleen tissues were isolated. The activities of mitochondrial complexes I, II and V were measured according to the method of Yoon et al [42].

### ATPase activity assay

The activities of ATP enzymes (Na^+^–K^+^–ATPase, Mg^2+^–ATPase and Ca^2+^–ATPase) in the chicken spleen were measured using commercial kits (Jiancheng Institute of Biotechnology, Nanjing, China) according to the manufacturer’s protocol. All samples were detected in duplicate in a single assay to avoid interassay variation.

### T-AOC, CAT activity, H_2_O_2_ production and ·OH inhibition assays

T-AOC, CAT activity, H2O2 production and ·OH inhibition assays were performed by comercial kits (Nanjing Jiancheng Bioengineering Institute, Nanjing, China) according to the manufacturer’s instructions.

### Real-time PCR

Total RNA was extracted from the spleen tissue by TRIzol reagent (Invitrogen, China). The RNA was reverse-transcripted to first-strand complementary DNA (cDNA) according to the manufacturer’s protocol (Invitrogen, China) and then stored at −80°C for PCR.

Primer Premier Software 5.0 (PREMIER Biosoft International, USA) was used to design specific primers of mitochondrial dynamics-related and apoptosis-related genes were designed by Primer Premier Software 5.0 (PREMIER Biosoft International, USA) (Table 1). β-actin was used as an internal reference. Quantitative real-time PCR (qPCR) reactions were run in a 20μL reaction mixture using an ABI 7500 Detection System (Applied Biosystems, USA). Each RT reaction was comprised of 0.4 μl of 50× ROX reference Dye II, 10 μl of 2× SYBR Green II PCR Master Mix (TaKaRa, China), 2 μl of cDNA, 0.4 μl of each primer (10 μM), and 6.8μl of PCR-grade water. The PCR procedure consisted of 95°C for 30 s, 40 cycles of 95°C for 5 s, and 60°C for 34 s. The mRNA relative abundance was determined via the method of Pfaffl [43].

**Table 1:** specific primers for mitochondrial dynamics-related and mitochondrial apoptosis pathway-related genes and β-actin

Gene	Forward primer	Reverse primer
β-actin	5-CCGCTCTATGAAGGCTACGC-3	5-CTCTCGGCTGTGGTGGTGAA-3
Mff	5-TGGGAAGGCTGAAGAGAGAA-3	5-GGTGTTCCCTCAAGTGTGGT-3
Drp1	5-GGCAGTCACAGCAGCTAACA-3	5-GCATCCATGAGATCCAGCTT-3
Opa1	5-GCTACGGACCAGGGTTATGA-3	5-GCTCAAGCATCCGTTGGTAT-3
Mfn2	5-TACCAGGCAGATTTCCATCC-3	5-GTGATTGCATTGGAACAACG-3
Mfn1	5-TGAGCATGTAGCAACGGAAG-3	5-AGCAAGCTGATTGACGGTCT-3
Bcl-xl	5- CTTTCAGCGACCTCACCTC -3	5- -TACCCATCCTCCGTTGTCCT -3
Bak	5- ACCCGGAGATCATGGAGA -3	5- GATGCCTTGCTGGTAGACG -3
Bax	5′-TATGGGACACCAGGAGGGTA-3′	5′-CGTAGACCTTGCGGATAAAGC-3′
p53	5′-CCCATCCTCACCATCCTTACA-3′	5′-CTCGATCTTGCGGTCCCTC-3′
Cyt-C	5’-AGGCAAGCACAAGACTGGA-3’	5’-CTGACTATCACCAAGAACCACC-3’
Caspase3	5-CATCT GCATCC GTGCCTGA-3	5-CTCTCGG CTGTGGTGGTGAA-3
Caspase9	5′-CCGAAGGAGCAAGCACG-3′	5′-AGGTTGGACTGGGATGGAC-3′

### Western blot analysis

Total protein was subjected to SDS-polyacrylamide gel electrophoresis under reducing conditions. The separated proteins were then transferred to nitrocellulose membranes using a tank transfer for 2 h at 200 mA in Tris–glycine buffer containing 20 % methanol. Membranes were blocked with 5 % skim milk for 24 h and incubated overnight with diluted primary antibody (Santa Cruz Biotechnologies, CA, USA). Followed by a horse-radish peroxidase (HRP) conjugated secondary antibody against rabbit IgG (Santa Cruz, USA). The membrane was incubated with a monoclonal β-actin antibody (Santa Cruz, USA), followed by incubation with a HRP conjugated goat anti-mouse IgG. The protein bands were visualized by enhanced chemiluminescence detection reagents (Applygen Technologies Inc., Beijing, China). The signal was detected by X-ray films (TransGen Biotech Co., Beijing, China). The optical density (OD) of each band was determined by an Image VCD gel imaging system (Beijing Sage Creation Science And Technology Co. Ltd., Beijing, China), and the relative abundance of the proteins were expressed as the ratios of OD of each of these proteins to that of β-action.

### Statistical analysis

Statistical analysis of all the data was performed with SPSS for Windows version 21.0; SPSS Inc., Chicago, IL. One-way analysis of variance followed by Tukey’s test was applied to analyze the descriptive statistics (mean values, standard deviation). *p* < 0.05 was considered to be statistically significant.
